# Supplementary material for: High-resolution characterization of gene function using single-cell CRISPR tiling screen
Source: Nat Commun. 2021 Jul 1;12:4063. doi: 10.1038/s41467-021-24324-0 (PMC8249386; doi:10.1038/s41467-021-24324-0)
Supplement: Supplementary file 1 — Supplementary Information [file 41467_2021_24324_MOESM1_ESM.pdf]

sc-Tiling: High-resolution characterization of gene function using single-cell CRISPR tiling screen

Yang et al.

Supplementary Figures 1 – 12
Supplementary Table 1 – 2

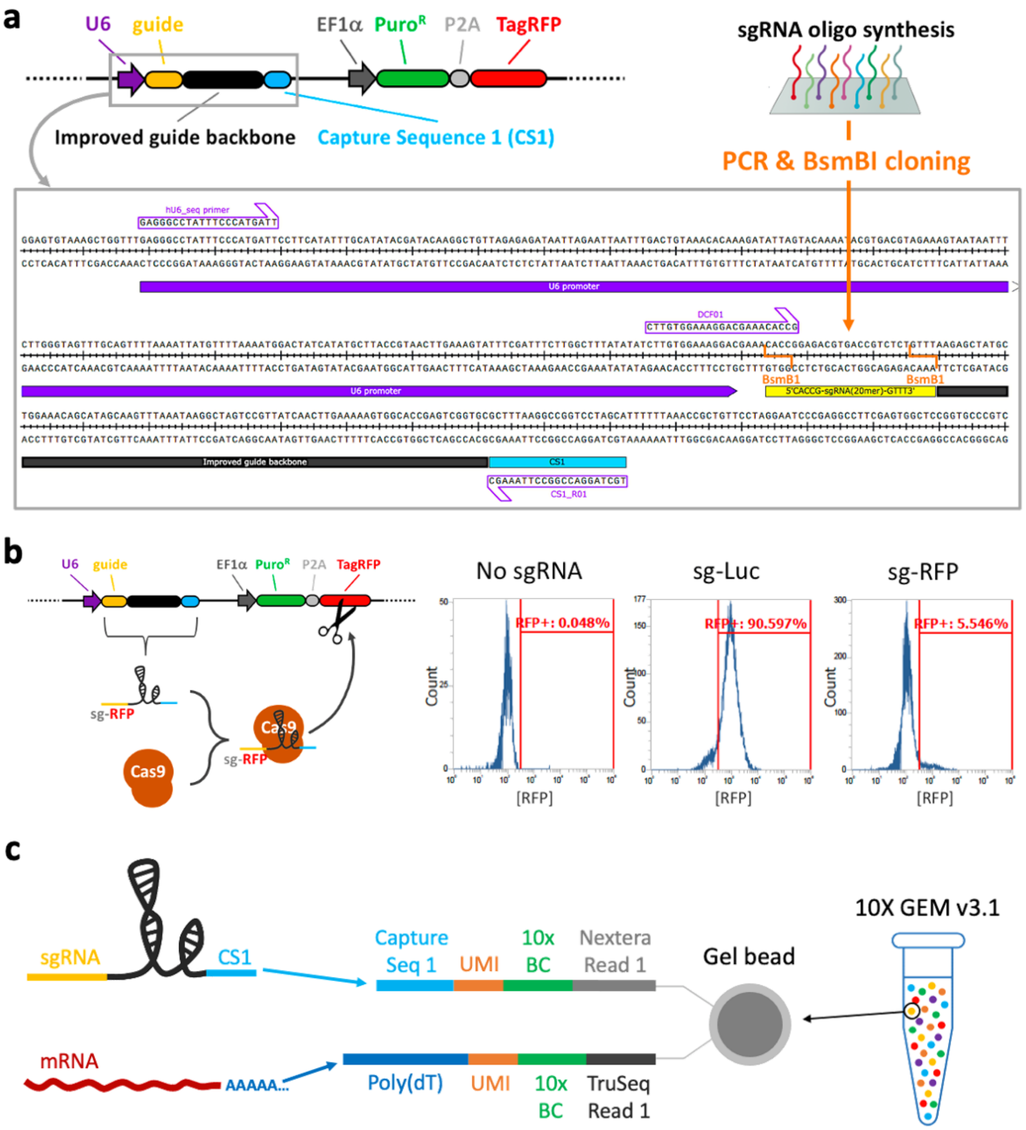

Supplementary Figure 1. Schematic outline of the single-cell CRISPR screen system. (a) Sequence map of the pUCS1EPR vector expressing a CS1-capturable sgRNA together with a puromycin-resistant gene (Puro^R) and a TagRFP fluorescent protein. Primers for Sanger (hU6_seq) and Illumina (DCF01 and CS1_R01) sequencing are listed. (b) Validation of CRISPR editing efficiency of the CS1-capturable sgRNA in Cas9-expressing cells using an RFP inactivation assay. (c) Cartoon illustration of the CS1 and poly(dT) capture mechanisms for single-cell CRISPR and mRNA sequencing.

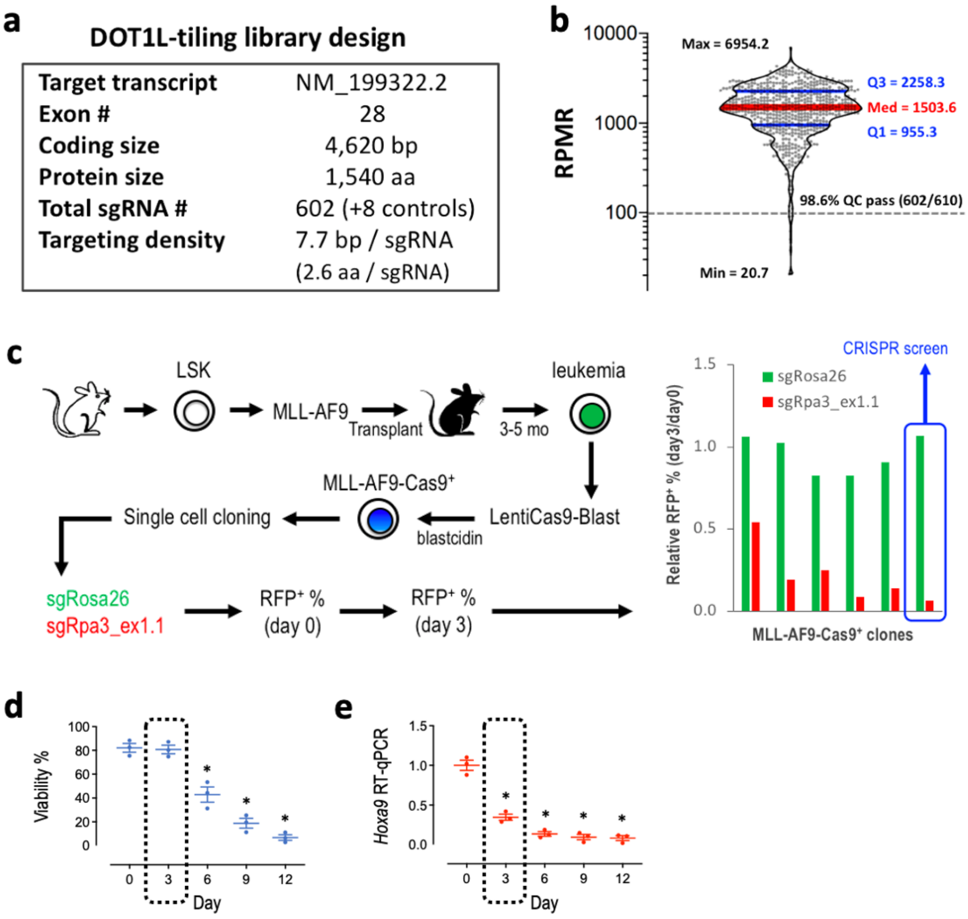

Supplementary Figure 2. CRISPR gene tiling screen in MLL-AF9 leukemia cells. (a) Specifications of the DOT1L-tiling CRISPR library targeting the coding exons of mouse *Dot1l*. (b) Distribution of individual sgRNA frequencies (reads per million reads; RPMR) in the DOT1L-tiling CRISPR library. 98.6 % of sgRNA passed the QC by exhibiting greater than 100 RPMR. (c) Establishment of monoclonal Cas9-expressing MLL-AF9 leukemia cells with high CRISPR editing efficiency. The sgRNA targeting *Rosa26* (green; negative control) and *Rpa3* (red; positive control) were reported by Shi et al.[1] (Supplementary Figure 3). (d) Cell viability and (e) *Hoxa9* (a DOT1L-driven gene) expression level of MLL-AF9-Cas9$^+$ cells transduced with a sgRNA targeting the KMT core. Data represent mean ± SEM of a triplicate experiment. *P < 0.01 by two-sided Student's t-test compared to day 0. Day 3 post-transduction (dotted box) was selected for sc-Tiling screen to avoid cell death signature influencing the transcriptomic analysis.

a

Poly(dT) captured mRNA library

Number of reads	185,270,701
Valid barcodes	96.2%
Q30 – Barcode	95.9%
Q30 – mRNA	92.7%
Q30 – Index	94.2%
Q30 – UMI	93.3%
Mean reads per cell	26,350
Median genes per cell	2,935
Total genes detected	15,921
Median UMI # per cell	11,341

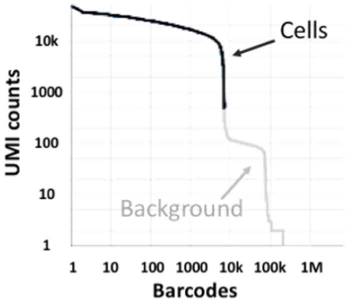

Alignment (mm10)

Genome	92.0%
Exons	76.0%
Introns	12.2%
Intergenic region	3.8%
Antisense	2.0%

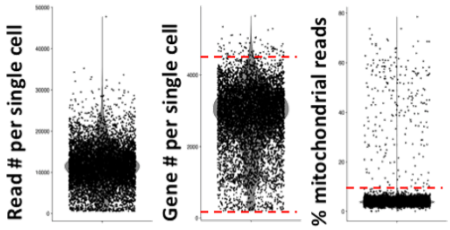

b

CS1 captured sgRNA library

Number of reads	8,910,124
Valid barcodes	84.2%
Q30 – Barcode	96.7%
Q30 – sgRNA	94.1%
Q30 – Index	91.7%
Q30 – UMI	96.7%
Mean reads per cell	1,267
Cells with 1 sgRNA	4,362
Cells with 2 sgRNA	527
Cells with 3+ sgRNA	54

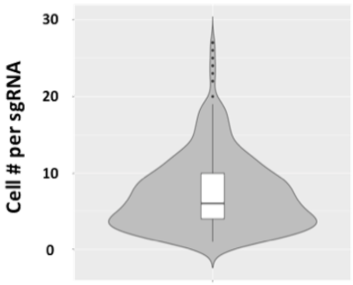

Supplementary Figure 3. Summary of sequencing specifications of sc-Tiling mouse *Dot1l* gene. Single-cell sequencing QC report of (a) poly(dT)-captured mRNA library and (b) CS1-captured sgRNA library. Box represents the 25th, 50th, and 75th percentiles, and the whisker indicates 1.5 interquartile range.

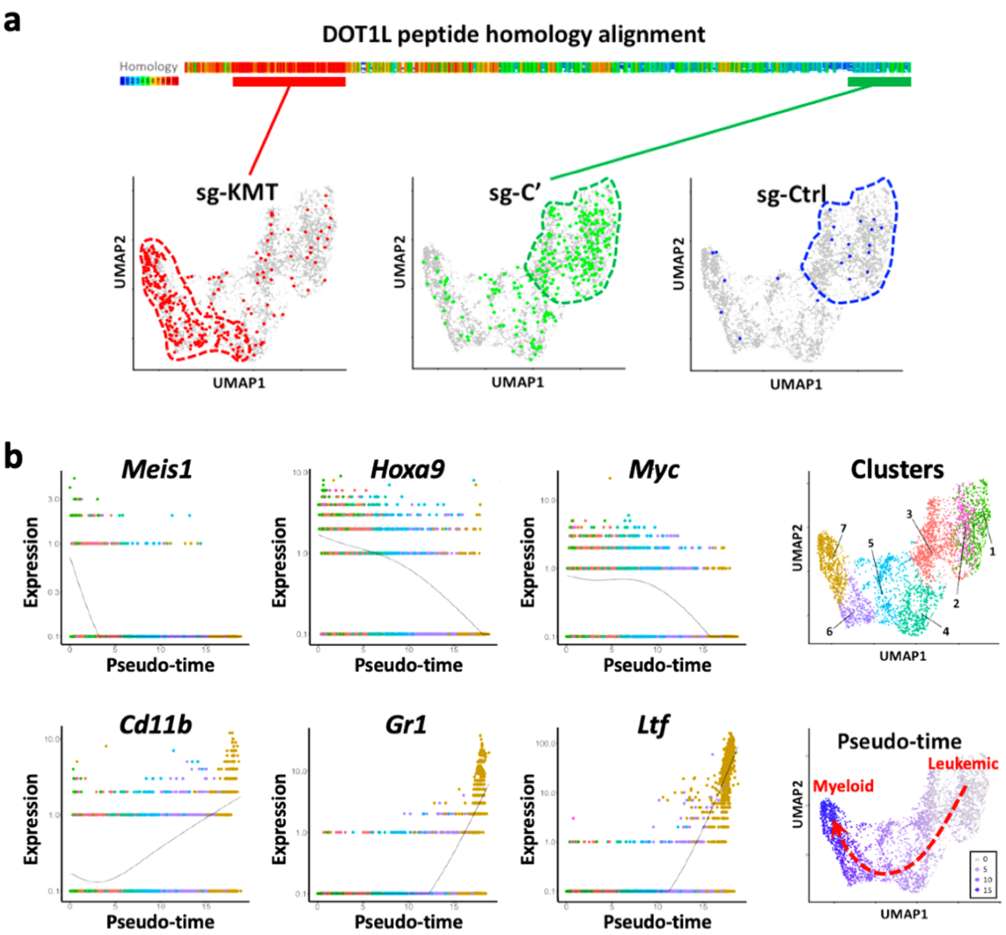

Supplementary Figure 4. Distribution and gene expression pattern of sc-Tiling single cells. (a) Distribution of single cells harboring sgRNAs targeting the KMT core (red; 56 sgRNAs), C-terminus (green; 54 sgRNAs), or negative controls (blue; 5 sgRNAs) on UMAP. Peptide sequence homology was analyzed using PRALINE multiple sequence alignment[2] at http://www.ibi.vu.nl/programs/pralinewww. (b) Association of the pseudo-time projection with the progressive reduction of leukemia-associated genes (*Meis1*, *Hoxa9*, *Myc*) or induction of myeloid-differentiation genes (*Cd11b*, *Gr1*, *Ltf*). Colored dots indicate single cells in each cell cluster (1–7) identified on UMAP (upper-right). Annotation of pseudo-time projection (bottom-right; purple gradient) indicates clustering of the leukemic (right) vs. myeloid (left) cells on UMAP.

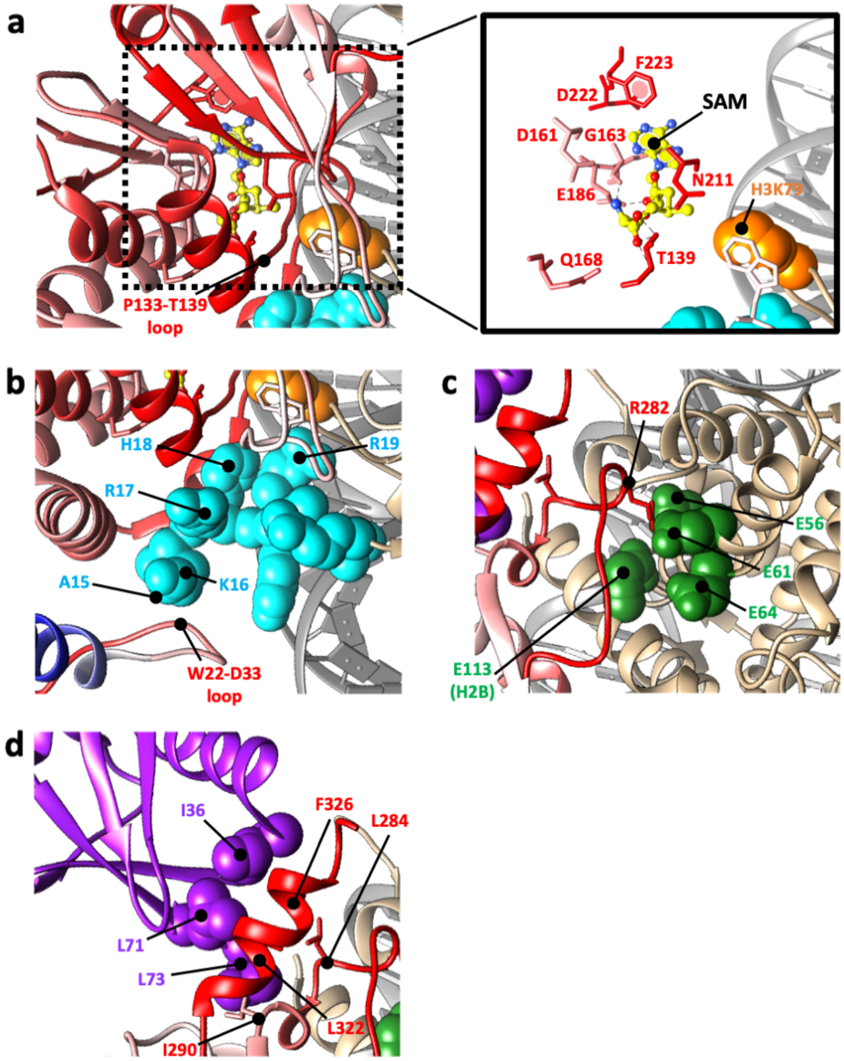

Supplementary Figure 5. Three-dimensional annotation of DOT1L sc-Tiling. Mapping of smoothed pseudo-time score to a cryo-EM structural model of "active state" DOT1L (residues M1–P332) bound to a ubiquitylated nucleosome (PDB ID: 6NQA)[3], as shown in Fig. 1H. (a) DOT1L catalytic pocket nearby histone H3K79 (orange spheres; methylation target of DOT1L) including the DOT1L residues directly interacting with the enzymatic substrate SAM (colored sticks). (b) DOT1L W22–D33 loop stabilizes the interaction with histone H4 N-terminal tail (cyan spheres; H4 residues A15–R19 are labeled). (c) DOT1L R282 loop interacts with an H2A/H2B acidic patch (green spheres; histone H2A E56/E61/E64 and H2B E113). (d) DOT1L T320–K330 helix (L322 and F326) and nearby residues (L284 and I290) interact with H2BK120 conjugated to ubiquitin (purple). The DOT1L contact points I36/L71/L73 on ubiquitin are labeled as purple spheres. Histones (gold; including H2A, H2B, H3, and H4) and DNA (grey) are shown.

a

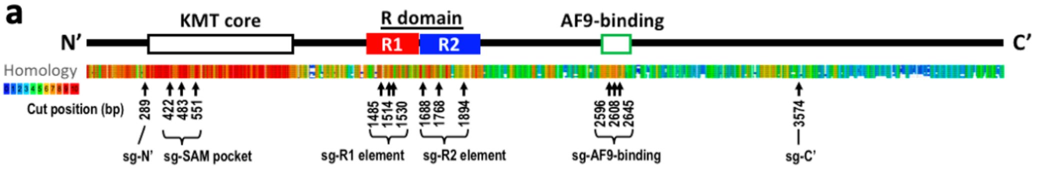

b

sgRNA name	Target gene	ID	sgRNA sequence	CRISPR cut position cDNA (bp)	CRISPR cut position Peptide (aa)
sg-N'	Mouse Dot1l	sg_mDot1l_289_a	GGCCGAGTATTCAGCTTCAT	289	97
sg-SAM pocket	Mouse Dot1l	sg_mDot1l_422_a	CTGGGCCACCAGGTCAAAGG	422	141
	Mouse Dot1l	sg_mDot1l_483_s	GATGACCTGTTTGTCGACCT	483	161
	Mouse Dot1l	sg_mDot1l_551_s	CTGCAAACATCACTACGGAG	551	184
sg-R1 element	Mouse Dot1l	sg_mDot1l_1485_a	TTGTATAAGCCAGGAACTGC	1485	495
	Mouse Dot1l	sg_mDot1l_1514_s	TACAAAGACCCCGCAGTACA	1514	505
	Mouse Dot1l	sg_mDot1l_1530_a	CCTGGTCCAGAAGCTGCTGT	1530	510
sg-R2 element	Mouse Dot1l	sg_mDot1l_1688_s	GACCTACAATGACCTGATTC	1688	563
	Mouse Dot1l	sg_mDot1l_1768_s	AGAAGGACAACAGCGAGCTT	1768	590
	Mouse Dot1l	sg_mDot1l_1894_s	GCCAGATCTCAGAGAAGCAG	1894	632
sg-AF9 binding	Mouse Dot1l	sg_mDot1l_2596_a	GTGCTGAGTGGAATGCTAAC	2596	866
	Mouse Dot1l	sg_mDot1l_2608_a	TTGGGCTGCACTGTGCTGAG	2608	870
	Mouse Dot1l	sg_mDot1l_2645_s	GCTGCCGGTCAGCATCCCAC	2645	882
sg-C'	Mouse Dot1l	sg_mDot1l_3574_a	CTGGGCTCATCCTCAGAGCC	3574	1192
sgLuc	Firefly Luciferase	sg_Luc_474	GATTCTAAAACGGATTACCA	-	-

c

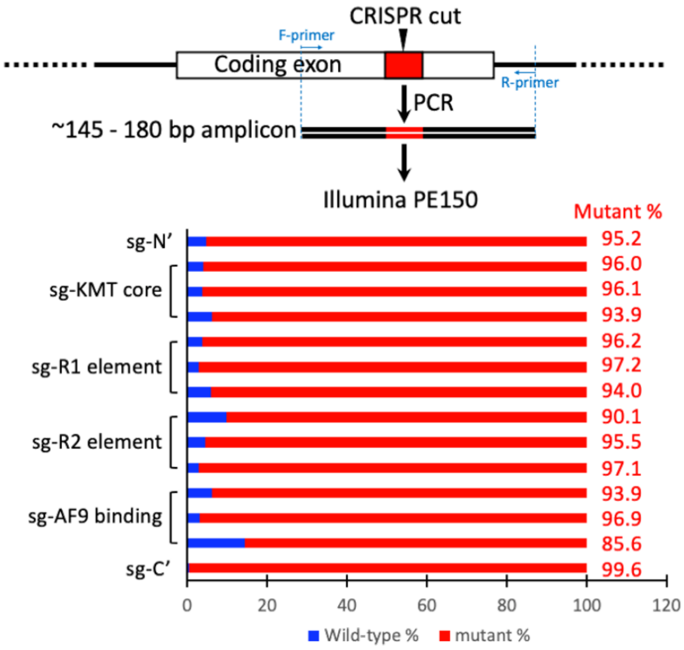

Supplementary Figure 6. Individual sgRNAs targeting mouse Dot1l coding regions for functional validation. (a) Schematic outline of the mouse DOT1L protein domains, peptide homology (by PRALINE multiple sequence alignment), and select sgRNA targeting positions. (b) Specifications of individual sgRNAs targeting indicated regions of mouse *Dot1l* and *Firefly luciferase*. (c) CRISPR editing efficiency of the selected sgRNAs (average 94.8 ± 3.4%) in MLL-AF9-Cas9^{+} cells at day 3 after sgRNA transduction.

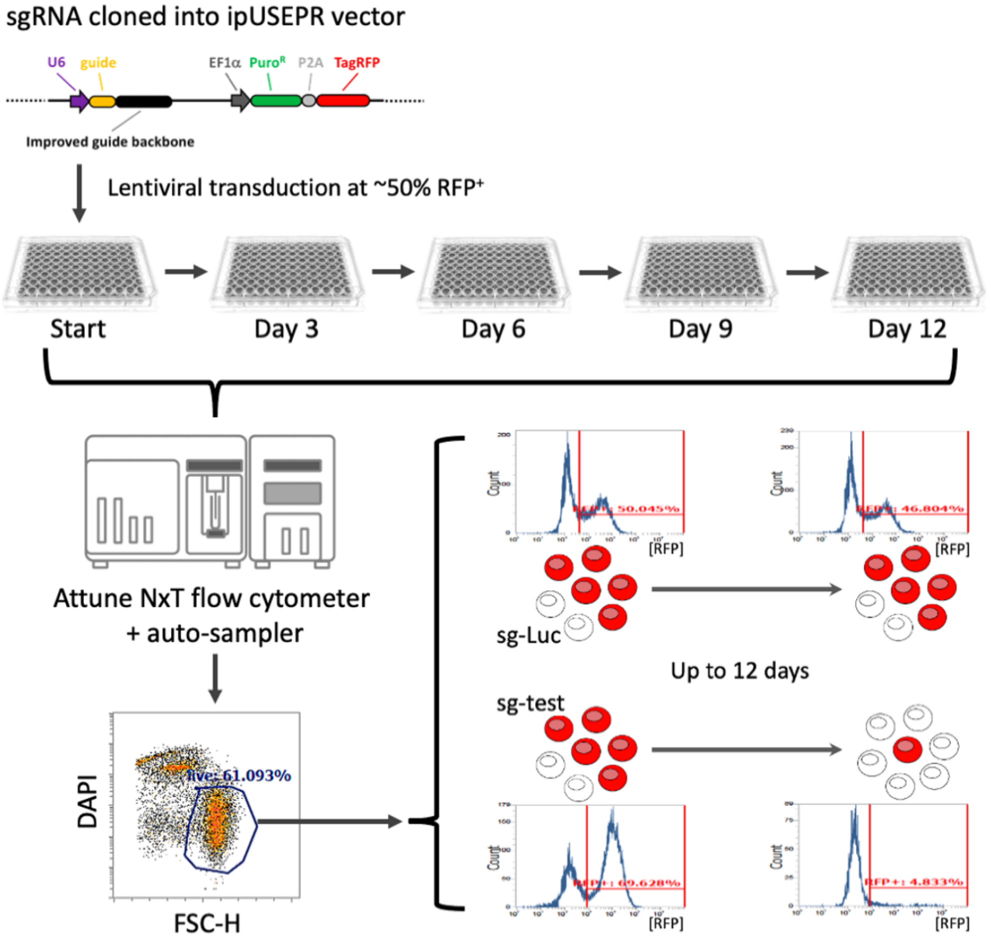

Supplementary Figure 7. Schematic outline of the RFP flow cytometric growth competition assay.

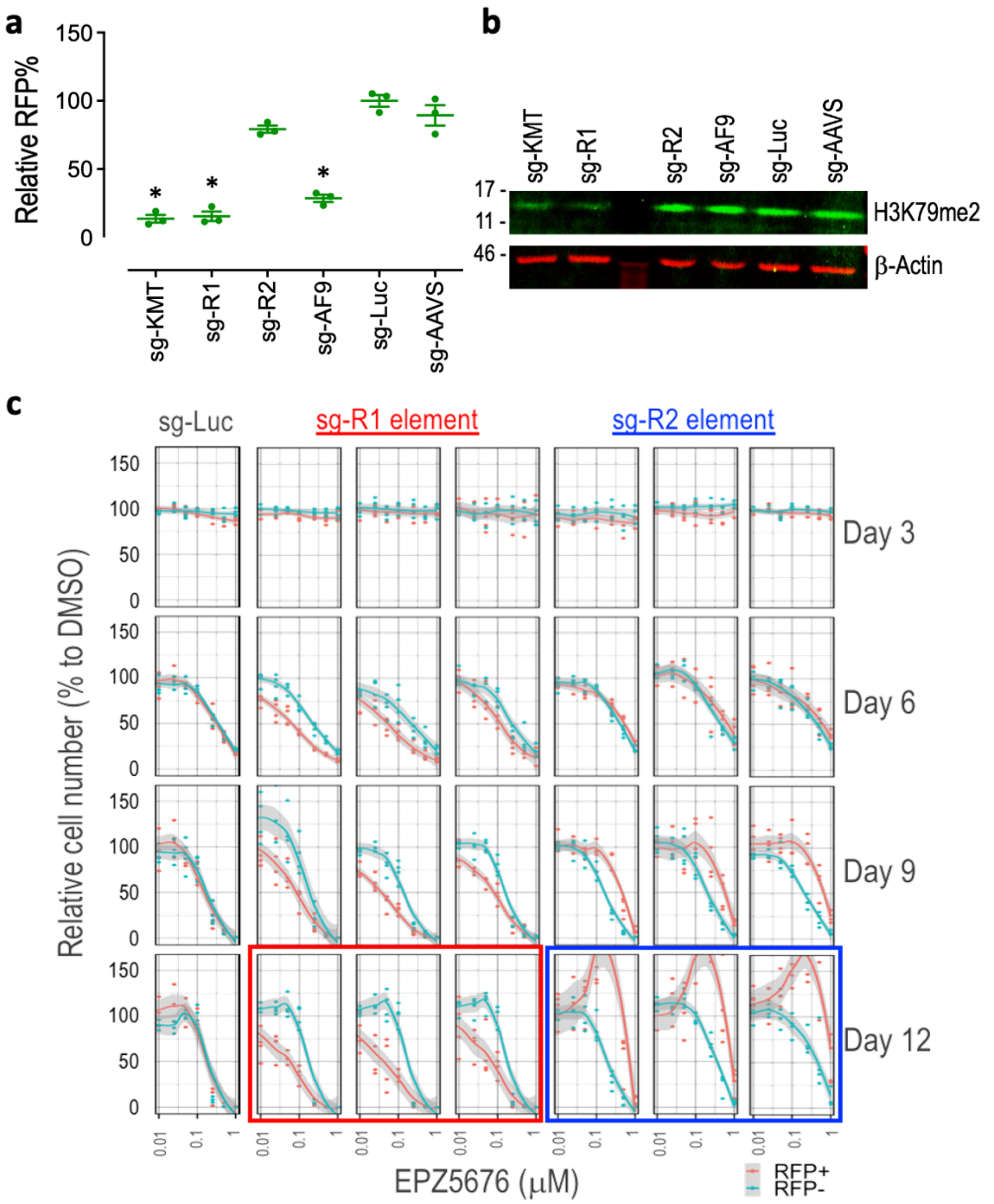

Supplementary Figure 8. Effect of individual sgRNAs targeting the R1 or R2 elements of DOT1L in response to EPZ5676 in MLL-AF9-Cas9^{+} leukemia cells. (a) Relative RFP% and (b) Western blot images of H3K79me2 (green) and β-Actin (red) in MLL-AF9-Cas9^{+} cells expressing indicated sgRNAs (one sgRNA per domain). A sgRNA targeting the AAVS safe-harbor locus (sg-AAVS; 5'-GGGGCCACTAGGGACAGGAT-3') performs similarly to sg-Luc. (c) Number of RFP^{+} (sgRNA transduced) and RFP^{-} (non-transduced) cells were measured by flow cytometer (Attune NxT, ThermoFisher). Data represent (a) mean ± SEM of a triplicate experiment. *P < 0.01 by two-sided Student's t-test compared to sg-Luc. (c) Observed values and mean ± 95% confidence interval of a quadruplicate experiment.

a

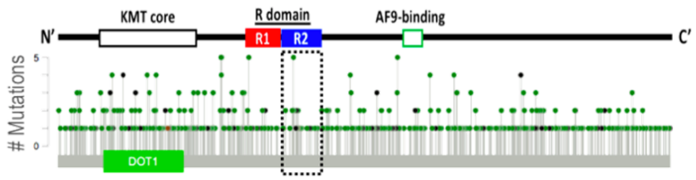

b

Protein Change	Wild type	Mutant
A567V	GCG	GTG
A573T	GCC	ACC
Q584P	CAG	CCG
R590C	CGC	TGC
A591T	GCG	ACG
A591V	GCG	GTG
R593C	CGC	TGC
R593H	CGC	CAC
G594D	GGC	GAC
G594S	GGC	AGC
K601M	AAG	ATG
C604Y	TGC	TAC
E605D	GAG	GAC
L609Q	CTG	CAG
D610Y	GAC	TAC
T613M	ACG	ATG
L626P	CTG	CCG
C637G	TGC	GGC
E646K	GAG	AAG

c

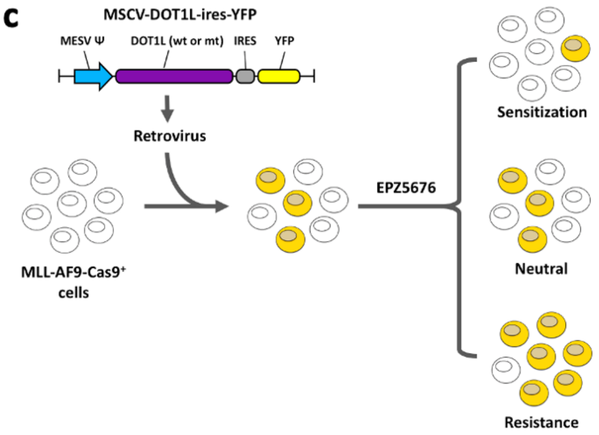

Supplementary Figure 9. Clinically observed *DOT1L* variant alleles in the R2 element. (a) A total of 779 missense *DOT1L* variants were identified in cBioPortal database (missense mutations labeled as green in the lolliplot). (b) Table shows a total of 19 missense variants observed in the R2 element (residues A558–C662). (c) Mutation of *DOT1L* cDNAs and schematic outline of the YFP flow cytometric growth competition assay.

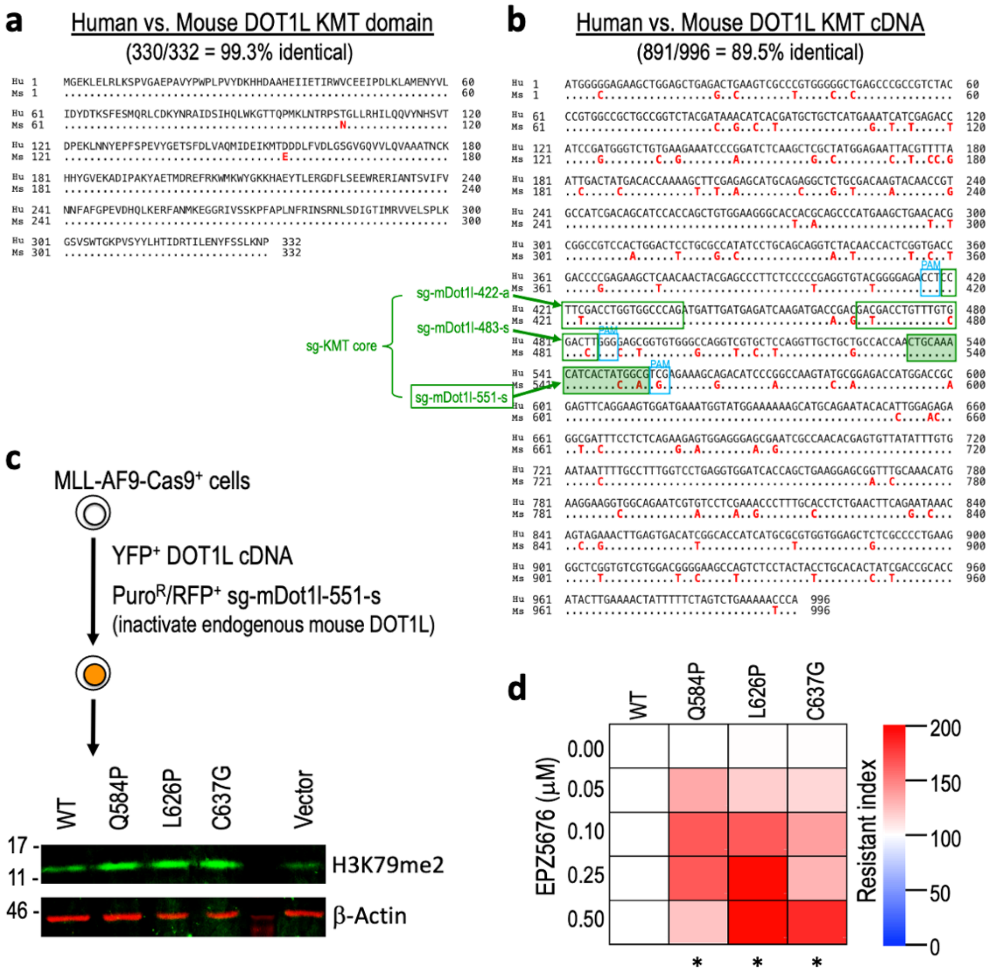

Supplementary Figure 10. Validation of drug-resistant *DOT1L* cDNA in MLL-AF9 cells missing an endogenous DOT1L activity. Comparison of (a) amino acid and (b) coding sequences between human and mouse DOT1L in the KMT domain (M1 – P332) showing highly conserved peptide (99.3% identical) but more degenerated DNA sequences (89.5% identical). One of the KMT core sgRNAs (sg-mDot1l-551-s) was predicted only targeting the endogenous mouse DOT1L (human *DOT1L* contains two nucleotide mismatches near the 3' end of sgRNA, plus one alternation in the PAM sequence). (c) Western blot images of H3K79me2 (green) and β-Actin (red) and (d) heatmap showing EPZ5676 resistant index of DOT1L-knockout MLL-AF9 leukemia cells transduced with wild-type (WT), Q584P, L626P, or C637G human *DOT1L* cDNA. Data represent the averaged values of a triplicate experiment. *Significantly ($P < 0.01$ by two-sided Student's t-test) higher resistance compared to wild-type at 0.5 μM EPZ5676.

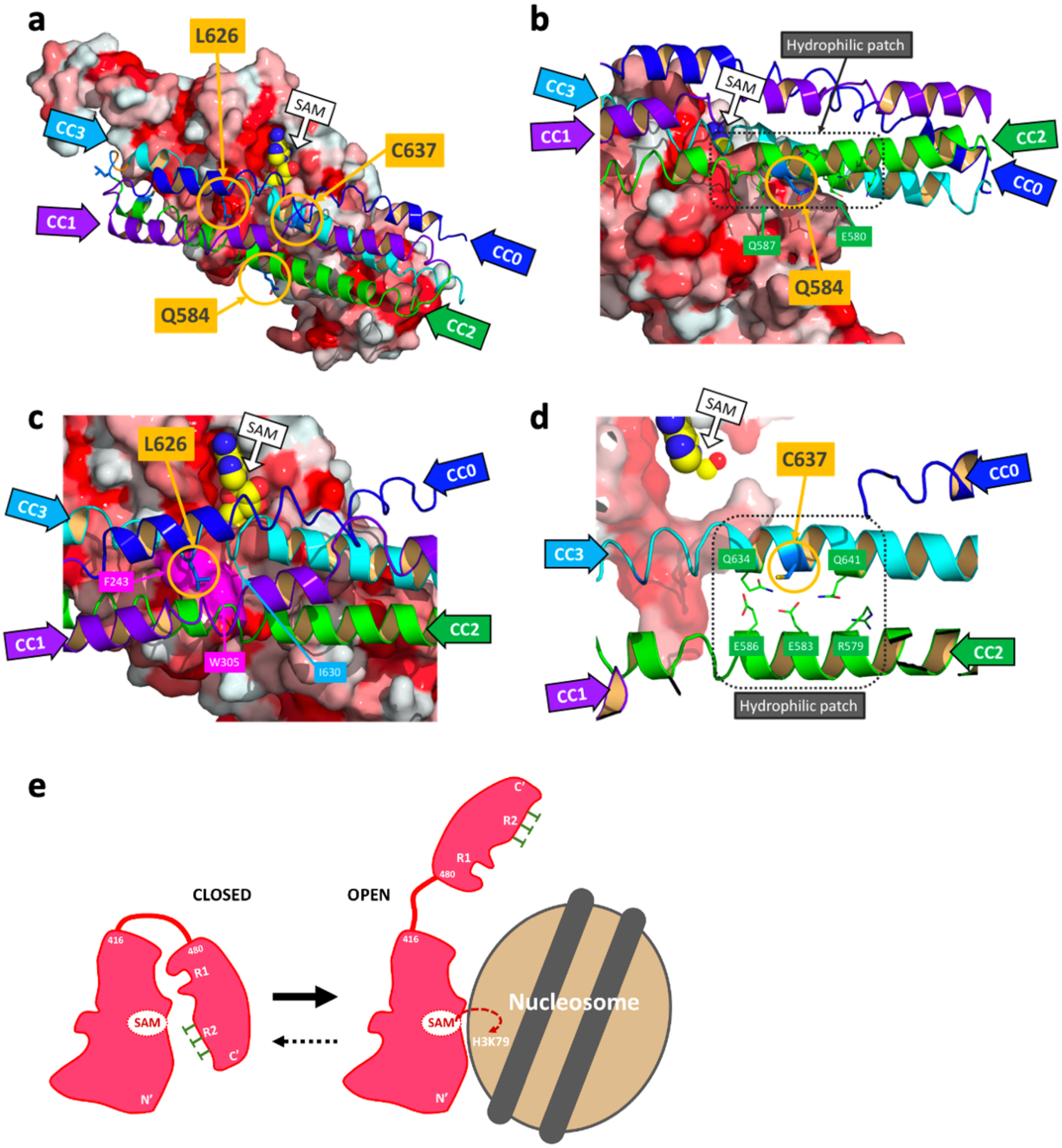

Supplementary Figure 11. Binding model of the DOT1L R domain with the KMT domain and drug-resistant mutations. (a) The DOT1L KMT domain (residues M1–P332; PDB ID 3QOW) is shown as surface with hydrophobic residues colored in red. The enzymatic substrate SAM is displayed as spheres. The R domain is depicted as a cartoon shown with CC0 (blue), CC1 (purple), CC2 (green), and CC3 (cyan) helices. The residues identified as drug-resistant mutations (Q584P, L626P, and C637G) in the R2 element (CC2 and CC3) are shown as colored sticks and their contact residues are displayed as lines. (b) Q584 is located in the center of a hydrophilic patch (dashed-line box) on both CC2 (Q576, Q577, R579, E580, Q581, E583, Q587, D588, N589) and CC3 (Q634, Q641) helices. Q584 stabilizes the CC2 helical structure via hydrophilic interactions with its neighboring residues E580 and Q587. Mutation of Q584 to a hydrophobic proline (Q584P), which will either break or kink the helix, will destabilize the CC2 helix structure and weaken the interaction between CC2 and CC3. (c) L626 is located in the middle of the CC3 helix and is a key residue by which the R domain interacts with the KMT domain (F243 and W305; shown as a pink patch) via hydrophobic interaction. It also stabilizes the CC3 helix by hydrophobic interaction

with I630. As proline tends to break helical structures, the L626P mutation may disrupt the CC3 helical structure and weaken the interaction between the R domain and KMT domain. (d) C637 is located in the center of a hydrophilic patch composed of Q634/C637/Q641 in CC3 and R579/E583/E586 in CC2 (dashed-line box) that stabilizes the helical structure and establishes the CC2/CC3 interaction. Mutation of C637 to a glycine (C637G) may disrupt the CC3 helical structure (due to the high conformational flexibility of glycine) and the interaction between CC2 and CC3. (e) Cartoon representation of the missense mutations (green) in the DOT1L R2 element, which favor the transition from a closed (left) to an open (right) state of the DOT1L KMT domain, thereby facilitating DOT1L-to-nucleosome interactions and H3K79 methylation.

a

Cell Line	Tissue Type Classification	Variant Classification	Reference Allele	Tumor Seq Allele	Protein Change
DND41	HAEMATOPOIETIC_AND_LYMPHOID_TISSUE	Silent	G	A	L578L
MFE319	ENDOMETRIUM	Silent	C	T	L559L
KARPAS45	HAEMATOPOIETIC_AND_LYMPHOID_TISSUE	Silent	G	A	E639E
NCIH513	PLEURA	Silent	C	G	L592L
RCCFG2	KIDNEY	Missense	G	A	**G594S**
PECAPJ34CLONEC12	UPPER_AERODIGESTIVE_TRACT	In_Frame_Del	ACA	-	N562del

b

Residue Variant	Variant ID	Location	Alleles	Evidence
**A567V**	rs1470399067	19:2213888	C/T	Frequency~gnomAD
**R590C**	rs371142941	19:2213956	C/T	Frequency~ESP~ExAC~TOPMed~gnomAD
**A591T**	rs776577348	19:2213959	G/A	Frequency~ExAC~TOPMed~gnomAD
**A591V**	rs376751030	19:2213960	C/T	Frequency~ESP~ExAC~TOPMed~gnomAD
**R593C**	rs200802307	19:2213965	C/T	Frequency~1000Genomes~ESP~ExAC~TOPMed~gnomAD
**R593H**	rs370589055	19:2213966	G/A	Frequency~ESP~ExAC~gnomAD
**G594S**	rs376032475	19:2213968	G/A	Frequency~ExAC~TOPMed~gnomAD
**T613M**	rs756254147	19:2214510	C/T	Frequency~ExAC~TOPMed~gnomAD

Supplementary Figure 12. Detection of cBioPortal observed DOT1L R2 variants in human cancer cell lines and population genomics databases. (a) DOT1L missense variant G594S reported in this study was also detected in Cancer Cell Line Encyclopedia (CCLE; BROAD Institute; total 1,457 human cell lines). (b) Eight out of 19 missense DOT1L variants discussed in this study were observed in the Single Nucleotide Polymorphism Database (dbSNP; including genomic information from 1000Genome, ExAC, TOPMed, GnomAD, GoESP) as potential germline variants in the human population.

Protein Change	Study	Sample ID	Cancer Type	Mutation Type	Allele Freq
A567V	Uterine Corpus E...	TCGA-B5-A3FC-01	Uterine Endometrioid Carcinoma	Missense	0.29
A573T	MSK-IMPACT Clini...	P-0010275-T01-IM5	Rectal Adenocarcinoma	Missense	0.15
Q584P	MSK-IMPACT Clini...	P-0006571-T01-IM5	Head and Neck Squamous Cell Ca...	Missense	0.15
R590C	Colon Adenocarci...	TCGA-CK-4951-01	Mucinous Adenocarcinoma of the...	Missense	0.15
A591T	Lung Adenocarcin...	TCGA-62-8395-01	Lung Adenocarcinoma	Missense	0.29
A591V	Stomach Adenocar...	TCGA-CG-5733-01	Stomach Adenocarcinoma	Missense	0.37
R593C	Colon Adenocarci...	TCGA-DM-A28F-01	Colon Adenocarcinoma	Missense	0.36
R593H	Colon Adenocarci...	TCGA-CM-4750-01	Colon Adenocarcinoma	Missense	0.58
G594D	MSK-IMPACT Clini...	P-0004051-T01-IM5	Colon Adenocarcinoma	Missense	0.29
G594S	MSK-IMPACT Clini...	P-0001851-T01-IM3	Adrenocortical Carcinoma	Missense	0.49
K601M	MSK-IMPACT Clini...	P-0009591-T01-IM5	Lung Adenocarcinoma	Missense	0.31
C604Y	MSK-IMPACT Clini...	P-0001448-T01-IM3	Pleomorphic Liposarcoma	Missense	0.12
E605D	Recurrent and Me...	P-0000573-T01-IM3	Oral Cavity Squamous Cell Carc...	Missense	0.20
L609Q	MSK-IMPACT Clini...	P-0000226-T01-IM3	Alveolar Rhabdomyosarcoma	Missense	0.44
D610Y	MSK-IMPACT Clini...	P-0010130-T02-IM5	Small Cell Lung Cancer	Missense	0.27
T613M	Head and Neck Sq...	TCGA-CV-7097-01	Head and Neck Squamous Cell Ca...	Missense	0.37
L626P	MSK-IMPACT Clini...	P-0007296-T01-IM5	Colon Adenocarcinoma	Missense	0.37
C637G	Lung Adenocarcin...	TCGA-69-7974-01	Lung Adenocarcinoma	Missense	0.06
E646K	MSK-IMPACT Clini...	P-0000138-T01-IM3	Breast Mixed Ductal and Lobula...	Missense	0.28

Supplementary Table 1. DOT1L R2 variants identified in cBioPortal database. In total, 19 missense variants were observed in cBioPortal (https://www.cbioportal.org).

Primer Name	Sequence	Tm (°C)
DCF01	CTTGTGGAAAGGACGAAACACCG	61
CS1_R01	TGCTAGGACCGGCCTTAAAGC	61

Supplementary Table 2. Primers for sgRNA sequencing.

Supplementary References

1 Shi, J. *et al.* Discovery of cancer drug targets by CRISPR-Cas9 screening of protein domains. *Nat Biotechnol* **33**, 661-667, doi:10.1038/nbt.3235 (2015).

2 Simossis, V. A. & Heringa, J. PRALINE: a multiple sequence alignment toolbox that integrates homology-extended and secondary structure information. *Nucleic Acids Res* **33**, W289-294, doi:10.1093/nar/gki390 (2005).

3 Worden, E. J., Hoffmann, N. A., Hicks, C. W. & Wolberger, C. Mechanism of Cross-talk between H2B Ubiquitination and H3 Methylation by Dot1L. *Cell* **176**, 1490-1501 e1412, doi:10.1016/j.cell.2019.02.002 (2019).
