# Supplementary material for: High-resolution characterization of gene function using single-cell CRISPR tiling screen
Source: Nat Commun. 2021 Jul 1;12:4063. doi: 10.1038/s41467-021-24324-0 (PMC8249386; doi:10.1038/s41467-021-24324-0)
Supplement: Supplementary file 4 — Reporting Summary [file 41467_2021_24324_MOESM4_ESM.pdf]

nature research

Corresponding author(s): Chun-Wei (David) Chen
Last updated by author(s): 05/31/2021

Reporting Summary

Nature Research wishes to improve the reproducibility of the work that we publish. This form provides structure for consistency and transparency in reporting. For further information on Nature Research policies, see our Editorial Policies and the Editorial Policy Checklist.

Statistics

For all statistical analyses, confirm that the following items are present in the figure legend, table legend, main text, or Methods section.

n/a	Confirmed	
[ ]	[x]	The exact sample size (n) for each experimental group/condition, given as a discrete number and unit of measurement
[ ]	[x]	A statement on whether measurements were taken from distinct samples or whether the same sample was measured repeatedly
[ ]	[x]	The statistical test(s) used AND whether they are one- or two-sided<br>*Only common tests should be described solely by name; describe more complex techniques in the Methods section.*
[x]	[ ]	A description of all covariates tested
[x]	[ ]	A description of any assumptions or corrections, such as tests of normality and adjustment for multiple comparisons
[ ]	[x]	A full description of the statistical parameters including central tendency (e.g. means) or other basic estimates (e.g. regression coefficient) AND variation (e.g. standard deviation) or associated estimates of uncertainty (e.g. confidence intervals)
[ ]	[x]	For null hypothesis testing, the test statistic (e.g. F, t, r) with confidence intervals, effect sizes, degrees of freedom and P value noted<br>*Give P values as exact values whenever suitable.*
[x]	[ ]	For Bayesian analysis, information on the choice of priors and Markov chain Monte Carlo settings
[x]	[ ]	For hierarchical and complex designs, identification of the appropriate level for tests and full reporting of outcomes
[ ]	[x]	Estimates of effect sizes (e.g. Cohen's d, Pearson's r), indicating how they were calculated

Our web collection on statistics for biologists contains articles on many of the points above.

Software and code

Policy information about availability of computer code

Data collection	Sequencing data was collected on NextSeq 550 and HiSeq (Illumina)
Data analysis	The computational codes/tool packages used in this study are available at https://github.com/l0yang05/singleCell_CRISPR_10x (GitHub) and through other developers and venders, including Genetic Perturbation Platform (Broad Institute), Seurat v3.0, Monocle, Gaussian kernel smoothing in R, CLC Main Workbench version 8.1 (QIAGEN), Bowtie2, edgeR R package 3.14.0, PSIPRED v3.3 server, MultAlin v5.4.1 server, I-TASSER server v5.1, ZDOCK v3.0.2 software, PyMOL v1.8.6 software (Schrödinger, LLC.), UCSF Chimera 1.15, ImageJ 1.8.0_172, PRALINE multiple sequence alignment, FlowJo v9, Attune NxT v3.1.2.

For manuscripts utilizing custom algorithms or software that are central to the research but not yet described in published literature, software must be made available to editors and reviewers. We strongly encourage code deposition in a community repository (e.g. GitHub). See the Nature Research guidelines for submitting code & software for further information.

Data

Policy information about availability of data

All manuscripts must include a data availability statement. This statement should provide the following information, where applicable:
- Accession codes, unique identifiers, or web links for publicly available datasets
- A list of figures that have associated raw data
- A description of any restrictions on data availability

The 10X Genomics single-cell CRISPR and RNA-seq data generated in this study have been deposited in the Gene Expression Omnibus (GEO) database under accession code GSE174307 (https://www.ncbi.nlm.nih.gov/geo/query/acc.cgi?acc=GSE174307). Three-dimensional protein structures (PDB ID 3UWP and 6NQA) were obtained from the Research Collaboratory for Structural Bioinformatics Protein Data Bank (RCSB PDB; https://www.rcsb.org). Consortium genomic information were obtained from cBioPortal (https://www.cbioportal.org), Cancer Cell Line Encyclopedia (CCLE; https://portals.broadinstitute.org/ccle), dbSNP

(https://www.ncbi.nlm.nih.gov/snp/) databases. Additional data that support the findings of this study are provided in the Supplementary Information/Source Data file. Source data are provided with this paper.

Field-specific reporting

Please select the one below that is the best fit for your research. If you are not sure, read the appropriate sections before making your selection.

- [x] Life sciences
- [ ] Behavioural & social sciences
- [ ] Ecological, evolutionary & environmental sciences

For a reference copy of the document with all sections, see nature.com/documents/nr-reporting-summary-flat.pdf

Life sciences study design

All studies must disclose on these points even when the disclosure is negative.

Sample size

Figure 1 and 2a,f-h: n = 1; one 10X Genomics experiment with 4,943 single cell data collected. This is an explorational trial that no sample size calculation was predetermined.
Figure 2b: Each data contains read counts of 610 sgRNAs (sequence shown in Figure S3). Sample size was determined based on the maximal density of the sgRNAs targeting the Dot1l coding regions.
Figure 2c: total 1,511 amino acid positions (dots). Sample size was determined by the total amino acid positions available in the DOT1L protein.
Figure 2d, 3c,d,h: n = 4 for each condition was required to have 80% power in detecting a minimal 50% difference at a statistical significance of $p<0.05$ using 2-tailed t-test analysis with an assumed standard deviation of 20% per group.
Figure 2g: total 3,299 genes observed. Sample size was determined based on the total gene number detected in the sc-RNAseq.
Figure 2h: total 101 genes. Sample size was determined based on the total gene number detected in the sc-RNAseq.
Figure 3a,b: Each data contains read counts of 610 sgRNAs (sequence shown in Figure S3). Sample size was determined based on the maximal density of the sgRNAs targeting the Dot1l coding regions.
Figure 3j: n = 1 for each condition based on the densitometry measurement from Figure 3i. No sample size calculation was predetermined.

Data exclusions

No data point was excluded.

Replication

Figure 1 and 2a,f-h: one 10X Genomics experiment with 4,943 single cells. No replicate was performed.
Figure 2b: n = 1 for each time point (d3, d6, d9, d12). Similar experiments were repeated in Fig 3 for d12 (n = 3)
Figure 2c: total 1,511 amino acid positions (dots). No replicate was performed.
Figure 2d, 3c,d,h: n = 3 independent sgRNAs for each domain.
Figure 2g,h: one 10X Genomics experiment with 4,943 single cells. No replicate was performed.
Figure 3a,b: n = 3 independently transduced cultures for each condition (control, EPZ5676). Similar experiments were repeated in Fig 3 for d3, d6, d8, d12 (n = 1 each).
Figure 3j: n = 1 for each condition based on the densitometry measurement from Figure 3i. Similar experiments were performed in Suppl Fig. 11c.

Randomization

In each experiment, a parental culture of MLL-AF9-Cas9+ cells was split into individual cultures randomly with an equal seeding density. Each culture received a sgRNA, cDNA, or CRISPR library without predetermination.

Blinding

The researchers were aware of the treatment conditions while executing and analyzing the experiments.

Reporting for specific materials, systems and methods

We require information from authors about some types of materials, experimental systems and methods used in many studies. Here, indicate whether each material, system or method listed is relevant to your study. If you are not sure if a list item applies to your research, read the appropriate section before selecting a response.

Materials & experimental systems

n/a	Involved in the study	
	x	Antibodies
	x	Eukaryotic cell lines
x		Palaeontology and archaeology
x		Animals and other organisms
x		Human research participants
x		Clinical data
x		Dual use research of concern

Methods

n/a	Involved in the study	
x		ChIP-seq
	x	Flow cytometry
x		MRI-based neuroimaging

Antibodies

Antibodies used

Rabbit anti-H3K79me2 antibody (Cell Signaling Technology; cat# 5427S; clone: D15E8; 1:1,000)

	Mouse anti-beta Actin antibody (Abcam; cat# ab8226; clone: mAbcam 8226; 1:1,000)<br>Rabbit anti-histone H3 antibody (Abcam; cat# ab1791: 1:10,000)<br>HRP-linked goat anti-rabbit IgG antibody (Cell Signaling Technology; cat# CST7074; 1:10,000)<br>Donkey anti-rabbit IgG antibody conjugated with Alexa Fluor 488 (Abcam; cat# ab150061;1:10,000)<br>Donkey anti-mouse IgG antibody conjugated with Cy3 (Sigma-Aldrich; cat# AP192C,; 1:10,000)
Validation	Rabbit anti-H3K79me2 antibody (clone D15E8) is a rabbit monoclonal antibody. A single band was detected by Western blot at ~15 kDa (corresponding to histone H3 molecular weight). The Western signal is decreased in cell samples treated with EPZ5676 (H3K79 methyltransferase DOT1L inhibitor) and CRISPR induced DOT1L knockout.<br>Mouse anti-beta Actin antibody (clone mAbcam 8226) is a mouse monoclonal antibody. A single band was detected by Western blot at ~42 kDa (corresponding to beta actin molecular weight).<br>Rabbit anti-histone H3 antibody (ab1791) is a rabbit polyclonal antibody. A single band was detected by Western blot at ~15 kDa (corresponding to histone H3 molecular weight).

Eukaryotic cell lines

Policy information about cell lines

Cell line source(s)	Mouse MLL-AF9 leukemic cells were generated by transformation of mouse bone marrow Lin−Sca1+cKit+ (LSK) cells with a MIG (MSCV-IRES-GFP) retrovirus expressing MLL-AF9 fusion protein and transplanted into sublethally irradiated recipient mice as previously described (Bernt et al, 2011; Chen et al, 2015). Cas9 expressing MLL-AF9 cells were established through lentiviral transduction of LentiCas9-Blast followed by Blasticidin S selection, single-cell cloning, and CRISPR editing efficiency test (Figure S2c).<br>HEK293 cells was obtained from ATCC.
Authentication	Cas9-expressing MLL-AF9 leukemic cells were characterized by (1) expression of GFP, (2) response to genetic/ pharmacological inhibition of DOT1L (Bernt et al, 2011; Chen et al, 2015) and (3) CRISPR gene editing activity (Figure S2c).<br>HEK293 cells was authenticated by ATCC (Karyotyping: der(1)t(1;15) (q42;q13), der(19)t(3;19) (q12;q13), der(12)t(8;12) (q22;p13)).
Mycoplasma contamination	Plasmocin was added in all culture medium to prevent mycoplasma contamiation. All cell lines tested negative for mycoplasma contamination using a Mycoplasma PCR Detection Kit (Abm cat# G238).
Commonly misidentified lines<br>(See ICLAC register)	No commonly misidentified cell lines were used in this study.

Flow Cytometry

Plots

Confirm that:

- [x] The axis labels state the marker and fluorochrome used (e.g. CD4-FITC).
- [x] The axis scales are clearly visible. Include numbers along axes only for bottom left plot of group (a 'group' is an analysis of identical markers).
- [x] All plots are contour plots with outliers or pseudocolor plots.
- [x] A numerical value for number of cells or percentage (with statistics) is provided.

Methodology

Sample preparation	Cas9-expressing MLL-AF9 cells were virally transduced with the designated constructs (RFP+ lentiviral sgRNA or YFP+ retroviral DOT1L variant cDNA) in 96-well plates at ~50% infection and monitored using flow cytometry for RFP or YFP (FP). At each time point, the live cell counts and the percentage of FP+ cells (FP%) were obtained using high-throughput flow cytometry and 4',6-diamidino-2-phenylindole (DAPI) dye exclusion.
Instrument	Attune NxT flow cytometer with autosampler (ThermoFisher).
Software	FlowJo (Becton, Dickinson & Company), Attune NxT flow software (ThermoFisher).
Cell population abundance	RFP+ (%) and YFP+ (%) cells were measured for the live cells (DAPI-negative).
Gating strategy	RFP-negative gate was defined by cells without an RFP transduction.<br>YFP-negative gate was defined by cells without a YFP transduction.<br>DAPI-negative gate was defined by cells without DAPI stain.

- [x] Tick this box to confirm that a figure exemplifying the gating strategy is provided in the Supplementary Information.
