# Supplementary material for: High-resolution characterization of gene function using single-cell CRISPR tiling screen
Source: Nat Commun. 2021 Jul 1;12:4063. doi: 10.1038/s41467-021-24324-0 (PMC8249386; doi:10.1038/s41467-021-24324-0)
Supplement: Supplementary file 5 — Description of Additional Supplementary Files [file 41467_2021_24324_MOESM5_ESM.pdf]

Title: Supplementary Data 1.
Description: Sequence of individual sgRNAs in the DOT1L-tiling CRISPR library. sgRNAs are named as “sg_mDot1l_(cut position in cDNA)_(sense/antisense)”.
